# Intracranial Fusiform and Circumferential Aneurysms of the Main Trunk: Therapeutic Dilemmas and Prospects

**DOI:** 10.3389/fneur.2021.679134

**Published:** 2021-07-09

**Authors:** Yunbao Guo, Ying Song, Kun Hou, Jinlu Yu

**Affiliations:** Department of Neurosurgery, The First Hospital of Jilin University, Changchun, China

**Keywords:** intracranial fusiform and circumferential aneurysm, main trunk, open surgery, endovascular treatment, review

## Abstract

Intracranial fusiform and circumferential aneurysms (IFCAs), especially those located on the main trunk, are uncommon and difficult to manage. Currently, literature focused on IFCAs on the main trunk of cerebral arteries is lacking. The treatment of IFCAs is still under debate. Therefore, in this review, we further explore the treatment of this complicated entity. In addition, we also present some interesting cases. Based on the literature review and our experience, we found that IFCAs are often located in the vertebrobasilar system and that ruptured or large symptomatic IFCAs are associated with increased mortality and higher rebleeding rates. The treatment strategies for IFCAs can be classified as deconstructive and reconstructive methods via open surgery and/or endovascular treatment (EVT). Currently, EVT is a popular method and the main therapeutic choice. In particular, flow diversion has revolutionized the treatment of IFCAs. Parent artery occlusion (PAO) with or without revascularization may still be considered a suitable choice. Complex IFCAs that cannot be resolved by EVT can also be treated via open surgery with or without extracranial–intracranial bypass. Targeted embolization for the weak points of IFCAs is a temporary or palliative choice that is rarely used. In summary, despite complications, both surgical treatment and EVT are effective options for appropriately selected cases. Due to the development of endovascular implants, EVT will have better prospects in the future.

## Introduction

Based on morphology, intracranial fusiform and circumferential aneurysms (IFCAs) are arterial dilatations that are 1.5-fold the normal diameter and incorporate the entire artery (1). IFCAs account for 3–13% of intracranial aneurysms (2). IFCAs can occur anywhere in intracranial arteries. When located on the main trunk, IFCAs tend to grow at the vertebral artery (VA) and basilar artery (BA). IFCAs rarely occur in the middle cerebral artery (MCA) and the internal carotid artery (ICA) (3).

The clinical course of IFCAs of the main trunk varies; these aneurysms can be stable, present with ischemia or mass effects, and even rupture (4). Progressive IFCAs of the main trunk can be associated with increased mortality and higher rebleeding rates; intervention is needed for these lesions (5). Despite advances in endovascular treatment (EVT) and surgical techniques, IFCAs of the main trunk represent a real challenge (6, 7). Currently, IFCAs are only defined based on morphological characteristics, and understanding of the implied nature of IFCAs is very limited. Few data are available on IFCAs, and standard treatment protocols are lacking.

**Figure 1:**
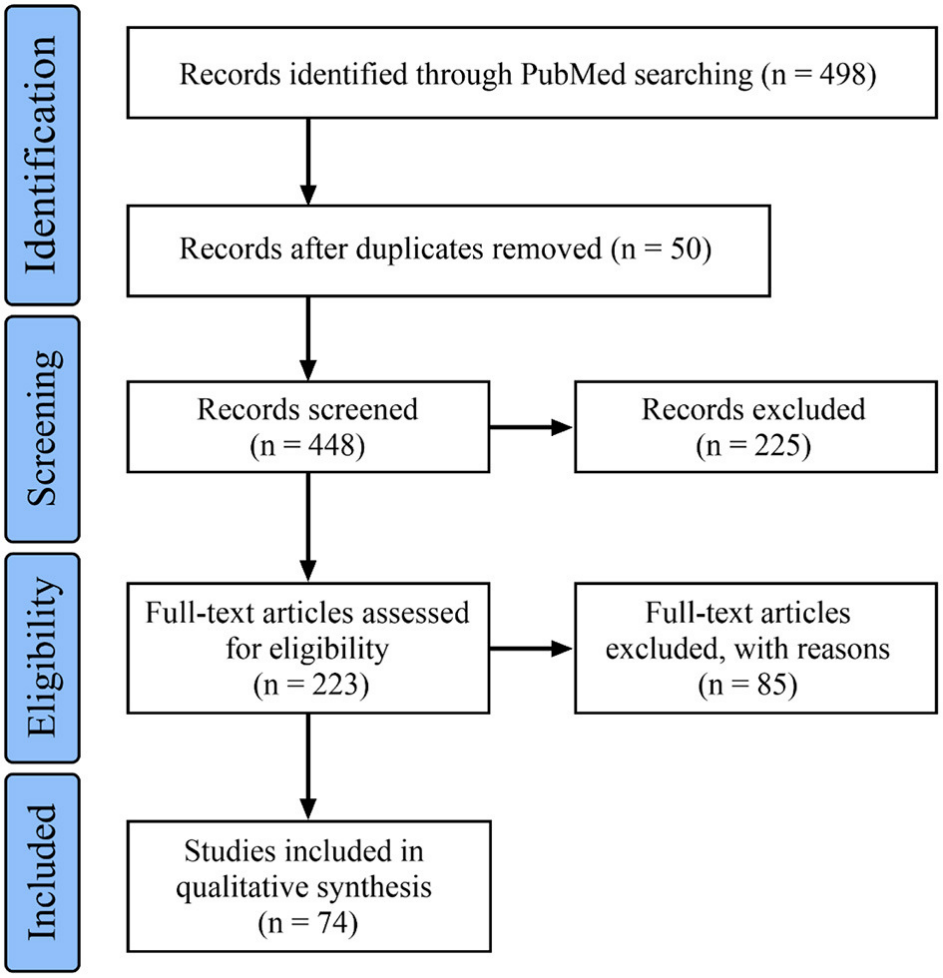
Flowchart of the search strategy.

In this article, “intracranial fusiform aneurysm,” “intracranial circumferential aneurysm,” and “intracranial large or giant dissection” were used as search terms to retrieve related literature from the PubMed database until May 13, 2021. In total, 74 references were cited, and the flowchart of the data search is shown in Figure 1. Then, a review is presented, mainly aiming to discuss the topic of therapeutic options for IFCAs of the main trunk. In addition, some interesting cases of IFCAs of the main trunk are provided to illustrate the clinical characteristics and therapeutic course.

## Clinical Characteristics

### Controversy of the Definition

When the fusiform aneurysm is short and similar to a sphere, it is called a circumferential aneurysm. Therefore, it is feasible to classify these aneurysms together as IFCAs.

Currently, a widely accepted IFCA definition exclusively based on morphology is lacking. Therefore, it is necessary to include multiple disease entities together to further assess IFCAs. The entities may include common dissection and arteriosclerotic dilation, fusiform dolichoectasia (which is characterized by dilated, elongated, and tortuous cranial arteries), and serpentine aneurysm (which is defined by special radiological characteristics presenting with a serpentine channel through the heavily thrombosed aneurysm) with a fusiform morphology (8, 9) because they are also treated with EVT (10, 11). In our review, broad definitions of involved IFCAs were noted, including the above entities

In addition, based on the clinical course, IFCAs can be classified as acute, such as dissecting aneurysms that typically cause subarachnoid hemorrhage or cerebral ischemia, and chronic, including aneurysms with relatively slow growth that may evolve into giant aneurysms, leading to serious complications (12, 13).

### Pathological Process

The pathogenesis of IFCAs is unknown, and the common proposed causes are dissection in youth and atherosclerosis in elderly individuals (10, 14–17). IFCAs are progressive lesions that often start with internal elastic lamina fragmentation and progress due to intramural hemorrhage (14, 18). IFCAs belong to type 3 of the Mizutani et al. classification, in which multiple dissections can lead to thrombus formation (19). Thus, IFCAs can grow and frequently produce mass effects, and frail aneurysms eventually rupture (20).

In IFCAs, the molecular mechanism plays an important role. For instance, somatic gain-of-function variants in the platelet-derived growth factor receptor β gene (PDGFRB) are mechanistically involved in the pathophysiology of IFCAs (21). In addition, Krüppel-like zinc-finger transcription factor 5 is highly expressed in large and giant unruptured cerebral aneurysms, including IFCAs (22).

### Natural History

Spontaneous regression of IFCAs is rare (23). Most unruptured chronic IFCAs may remain temporarily stable (24). In a report by Moon et al., 91.5% of asymptomatic IFCAs that were not too large proved to be stable within the first few years; however, those with lengths >6.9 mm were at risk of continuous growth (25). In a report by Sacho et al., non-atherosclerotic IFCAs remained stable unless symptomatic or >7 mm in diameter, and atherosclerotic IFCAs had a worse course (26). IFCAs with a diameter >10 mm may be more dangerous (20). In posterior circulation IFCAs, the natural history is worse (23, 27, 28). In particular, for ruptured or symptomatic acute IFCAs, the natural history is worse than that of chronic IFCAs, and acute IFCAs tend to rebleed or grow rapidly in a short time (29).

## Therapeutic Principle

Most asymptomatic IFCAs can be treated conservatively (30). When serial imaging indicates significant enlargement of IFCAs over time or IFCAs become symptomatic, aggressive intervention is necessary. In the meta-analysis by Nasr et al., conservative management for ruptured IFCAs resulted in a mortality rate of 38% after a mean follow-up period of 18 months (31). When IFCAs present with ischemia, anti-platelets should be temporarily administered to relieve the symptoms before surgical intervention (32).

The intervention should be tailored on a case-by-case basis determined by the location, size, configuration, being acute or chronic, availability of collateral flow, and risk of intervention (33). The surgeon must know clearly that some IFCAs cannot be treated or should be treated with caution, and the treatment should be limited to those patients who have a higher likelihood of benefitting from the therapy. Treatment should not be performed only because it is technically feasible; the aim should be to improve the clinical outcome of the patient.

If the IFCA requires intervention, multiple strategies could be chosen, including a deconstructive technique to occlude the IFCA, a reconstructive technique to restore the normal blood flow, and a combined reconstructive/deconstructive approach including both surgical and EVT choices (14, 34). For acute rupture or symptomatic IFCAs, prompt treatment is recommended, and deconstructive trapping has a definitive effect. For chronic IFCAs, the reconstructive technique is the first choice.

Currently, EVTs have emerged as the first-choice treatment, especially flow diversion (FD), which has revolutionized the treatment of IFCAs (35, 36). Open surgery is generally reserved for cases that cannot be treated with EVT. For some complex IFCAs, staged multimodality treatment is necessary and reasonable (37). However, the best therapeutic choice is controversial, and no definitive solution has been discovered because EVT and open surgery remain only partially successful in improving the devastating natural course.

## Fd Deployment

**Figure 2:**
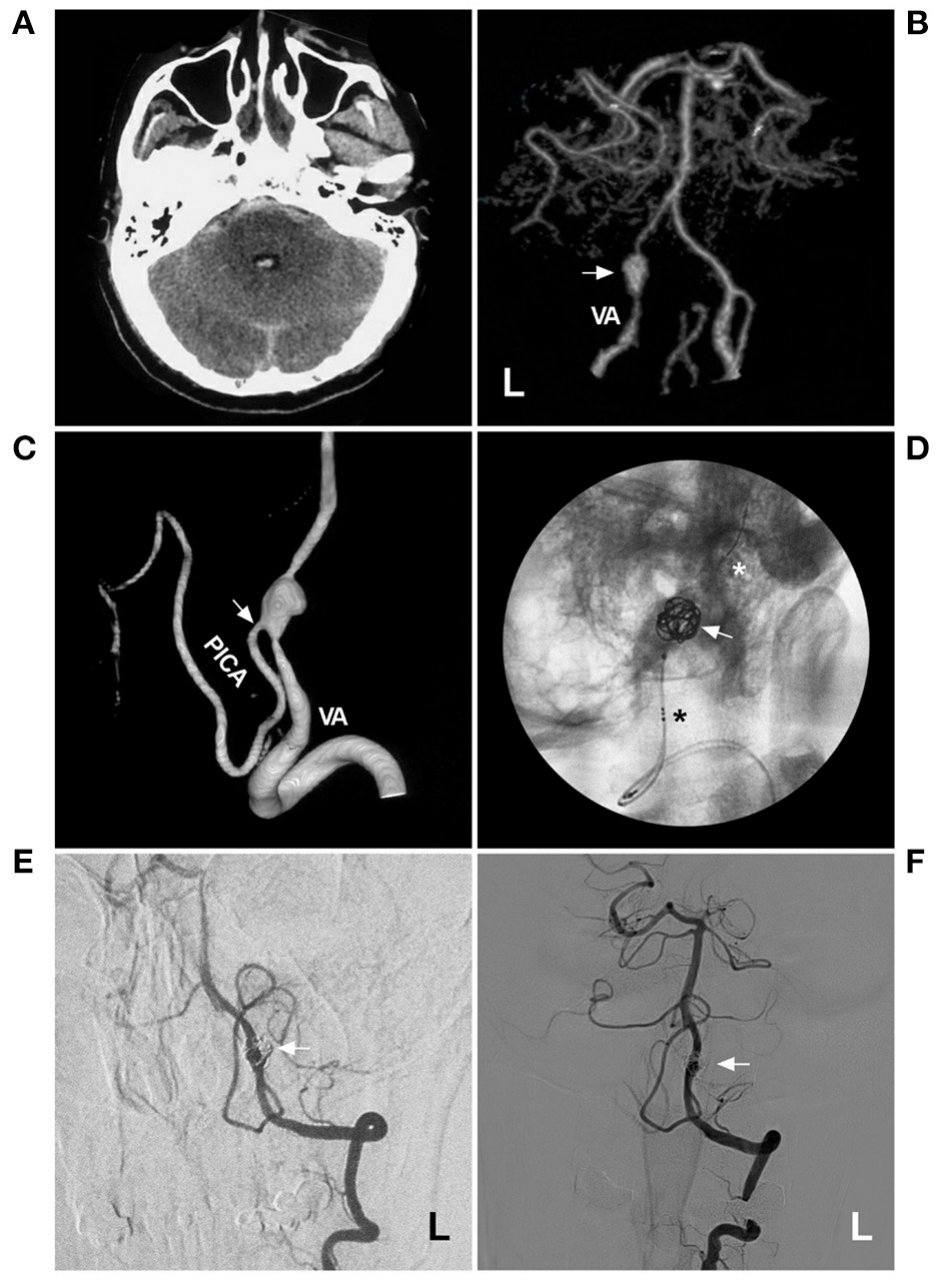
Coiling a VA acute fusiform aneurysm with stent assistance. **(A)** CT showed subarachnoid hemorrhage and fourth intraventricular hemorrhage. **(B)** CTA showed a fusiform aneurysm (arrow) on the left VA. **(C)** Three-dimensional DSA showed the PICA (arrow) from the aneurysm. **(D)** X-ray film showed the LVIS (asterisks indicate the proximal and distal markers) to assist in coiling the aneurysm. **(E,F)** 1-year **(E)** and 2-year **(F)** follow-up DSA of the left VA showed no recurrence of the aneurysm. CT, computed tomography; CTA, computed tomography angiography; DSA, digital subtraction angiography; L, left; PICA, posterior inferior cerebellar artery; VA, vertebral artery.

Regarding EVT before the era of FD, the placement of single, overlapping, or parallel low-metal coverage stents assisted with or without coiling was effective for IFCAs, especially for small IFCAs (38–40). Figure 2 shows the use of the conventional stent-assisted coiling technique in a patient. In this patient, the IFCA was a ruptured acute dissection that caused subarachnoid hemorrhage. Urgent EVT was needed, and the acute dissection was resolved.

Currently, FD with 24–55% metal coverage is a better option, and FD has revolutionized the treatment for unruptured chronic IFCAs and is used with caution in ruptured acute IFCAs (41, 42). After FD deployment, IFCAs begin to form thrombi; endothelialization subsequently occurs. The endothelium shrinks and collapses around the device over a period of 6–12 months with preservation of perforating branches. Finally, FDs are incorporated into the parent vessel (43, 44).

For the application of FD application for IFCAs, the use of telescoping FDs with 25–30% overlap is often required to cover the entire IFCA (45). In addition, overlapping FDs can be employed for a greater flow-diverting effect (46). For this purpose, the new-generation double-layered FD is more useful (47). However, this strategy must be used cautiously to avoid ischemia from perforator occlusion, especially in the BA or MCA (46, 48).

As a reconstructive technique, on rare occasions, it is necessary to combine FD deployment with the deconstructive approach. For instance, for large IFCAs of the vertebrobasilar junction, after FD deployment from the VA to BA, the contralateral VA needs to be occluded with coiling to reduce the endoleak from the contralateral blood flow (45, 49).

In theory, FD plus coiling may be helpful, especially for ruptured acute IFCAs, because adjunctive coils can act as scaffolds to reduce foreshortening of the FD. In addition, the coils may also protect against delayed aneurysm rupture as stasis promoters and result in higher rates of IFCA occlusion (50, 51). However, the effect of coiling assistance in FD deployment remains controversial in chronic IFCAs, and coiling does not prompt aneurysm occlusion (52).

The nature of IFCAs is associated with the effect and risk of FD deployment. If the common dissection and arteriosclerotic dilation are not too long, FD can be released easily, and the prognosis is good. However, for fusiform dolichoectasia, FD deployment is limited due to thrombosis and progressive growth, and the prognosis is uncertain (53).

**Figure 3:**
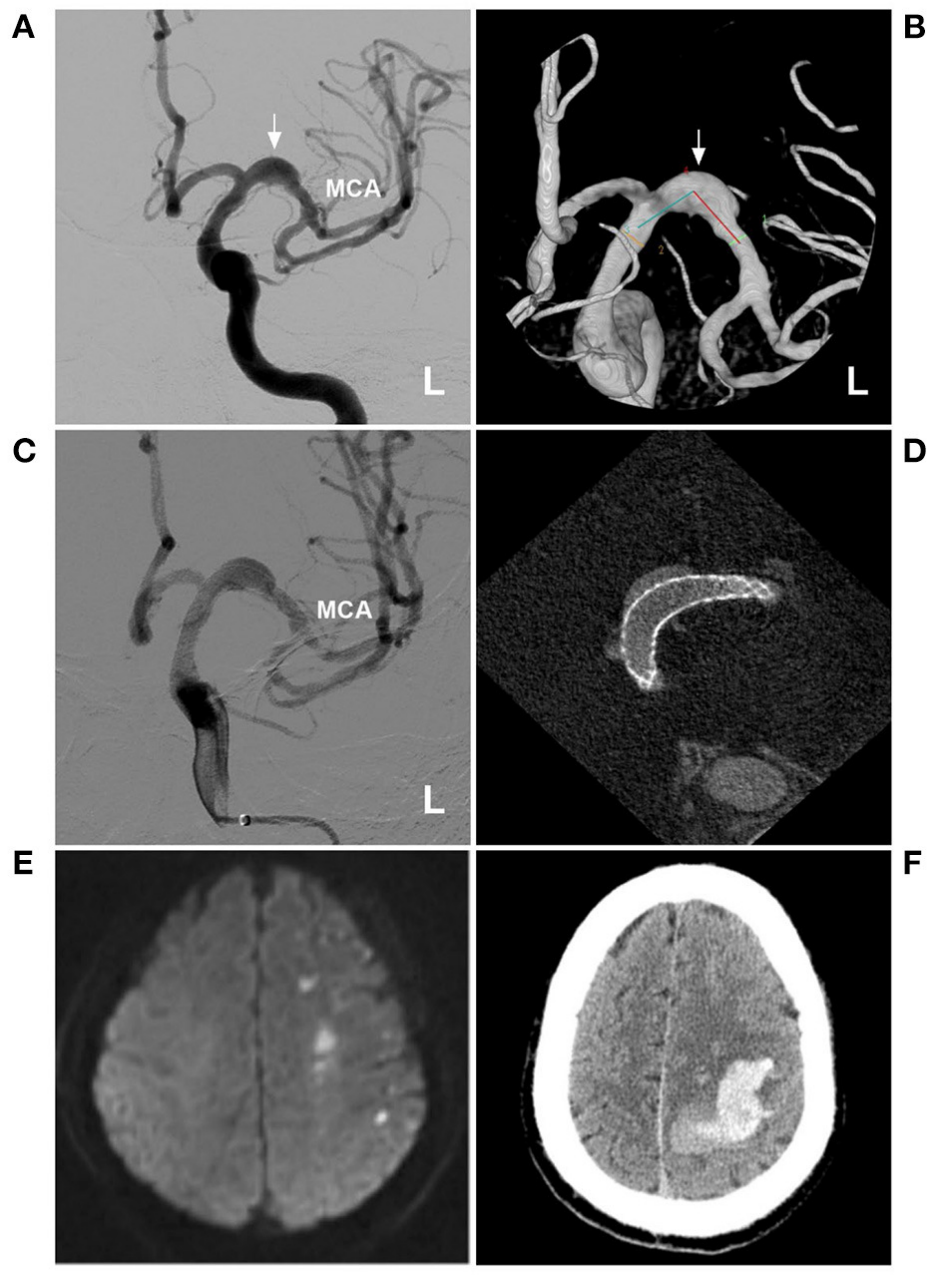
FD deployment for a fusiform aneurysm of the proximal MCA. **(A,B)** DSA **(A)** and three-dimensional DSA **(B)** showed a fusiform aneurysm of the proximal MCA (arrows). **(C,D)** DSA **(C)** and maximum-intensity projection image **(D)** showed FD deployment. **(E)** MRI showed acute infarction in the frontal and parietal lobes. **(F)** CT showed the delayed intracerebral hemorrhage. CT, computed tomography; DSA, digital subtraction angiography; FD, flow diversion; MCA, middle cerebral artery; L, left; MRI, magnetic resonance imaging.

A typical case treated with FD is shown in Figure 3. In this case with chronic asymptomatic IFCA at the beginning of the MCA without apparent perforating arteries, FD deployment was performed to prevent progressive growth. However, delayed remote hemorrhage occurred postoperatively. Thus, for small chronic dissections, interventions should be performed with caution.

## Parent Artery Occlusion

PAO in IFCA is a deconstructive technique, especially when combined with revascularization; this old technique is still currently being performed. Before PAO, neurologic symptoms should be carefully monitored during the balloon occlusion test (BOT) for at least 20 min after the administration of a bolus of heparin (54, 55). When the BOT is evaluated, venous-phase delay is the most important parameter. It is defined as the delay of opacification and assessed by the appearance of the first cortical vein in the territory of the PAO compared with that of its contralateral counterpart (56).

When venous filling is prolonged, BOT is not tolerated, and revascularization is necessary (57, 58). In the study by Shimizu et al., PAO with high-flow bypass was recommended for a venous-phase delay of >2 s. For a venous-phase delay of 1–2 s, the low-flow bypass of superficial temporal artery MCA was sufficient and recommended prior to PAO (56). Aggressive antithrombotic treatment was recommended before and after PAO (56).

PAO can be performed using balloons, coils, clipping, and Hunterian ligation at the proximal segment of the parent artery or trapping IFCA (59–62). The most definite effect of PAO was noted in the proximal and distal parent arteries in the trapping aneurysm, which is the most applicable to those arteries without branches from the acute IFCAs (63). Proximal PAO is also a good choice for chronic IFCAs. After this type of PAO, the blood flow between the “inflow zone” and “outflow zone” is reversed; the procedure is effective because the “outflow zone” is relatively risky and the “inflow zone” is most vulnerable (64). Ipsilateral PAOs are the most common, but bilateral PAOs are occasionally needed (65).

Although PAO can solve IFCA rupture or rerupture, the mass effect cannot be resolved, and IFCAs may even become enlarged due to thrombosis (66). At this time, intra-aneurysmal thrombectomy may be required (67, 68).

**Figure 4:**
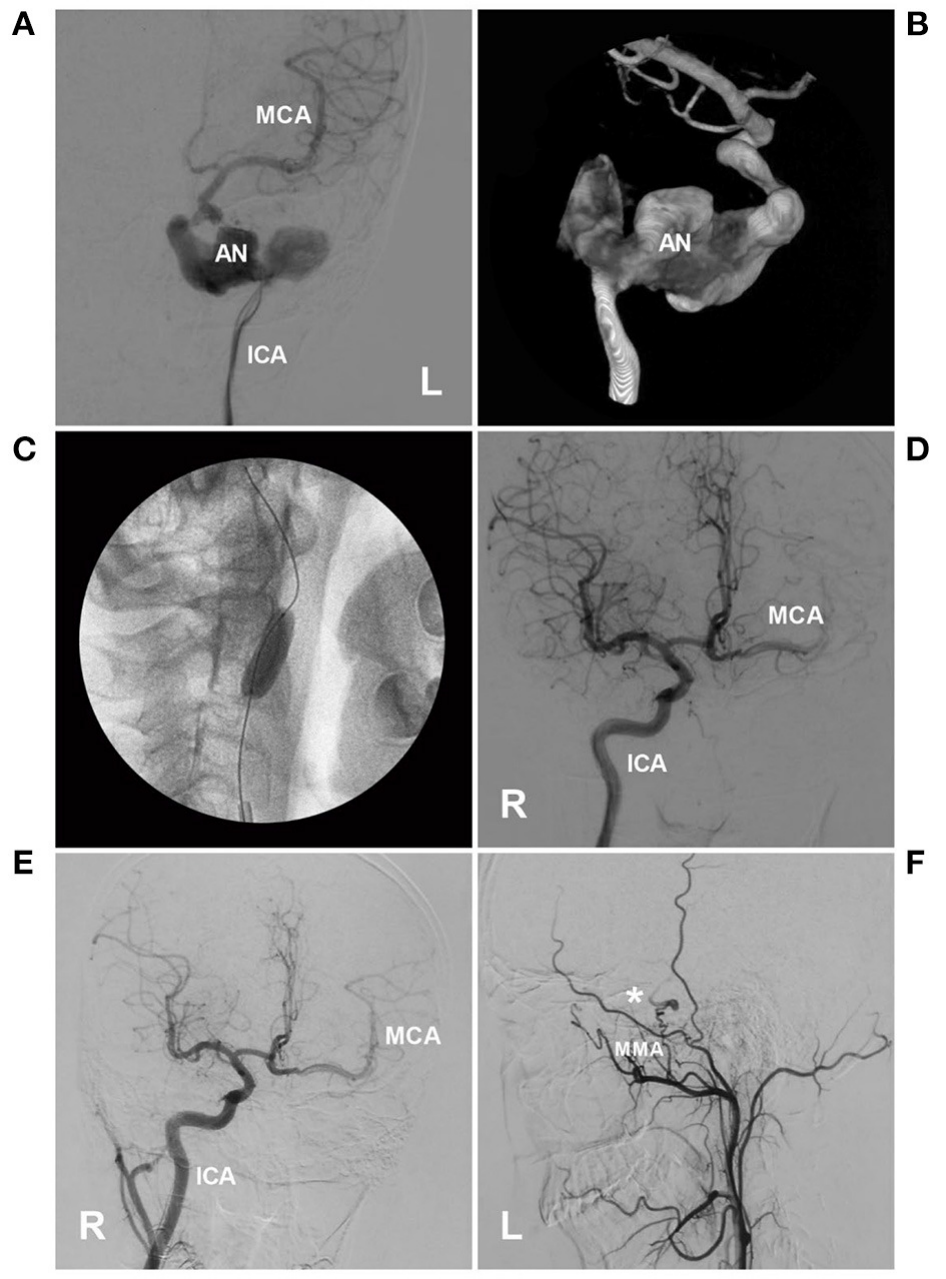
PAO of the ICA for a giant petrous segment aneurysm. **(A,B)** Left DSA **(A)** and three-dimensional DSA **(B)** showed an unregular giant aneurysm (AN) on the petrous segment of the ICA. **(C)** X-ray film showed that the BOT was performed, and the balloon was located in the ICA under the aneurysm. **(D)** During the BOT, DSA of the right ICA showed collateral circulation through the AcomA to the left MCA. **(E)** After PAO of the left ICA, 1-year follow-up DSA showed that collateral circulation through the AcomA was sufficient. **(F)** The external carotid artery showed anastomosis between the MMA (asterisk) and intracranial pial artery. AcomA, anterior communicating artery; BOT, balloon occlusion test; DSA, digital subtraction angiography; ICA, internal carotid artery; L, left; MCA, middle cerebral artery; MMA, middle meningeal artery; PAO, parent artery occlusion; VA, vertebral artery.

**Figure 5:**
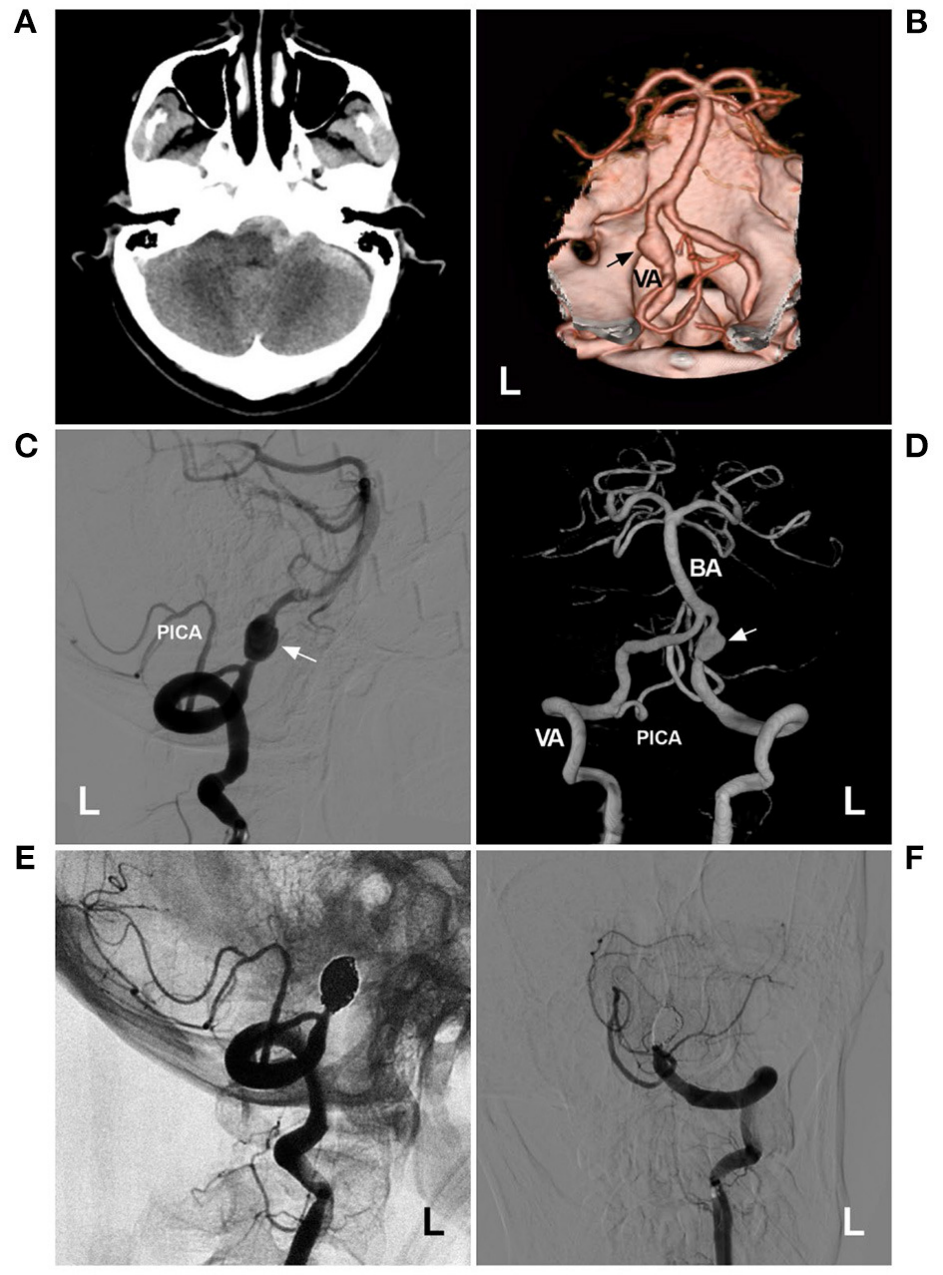
PAO for a VA acute fusiform aneurysm. **(A)** CT showed subarachnoid hemorrhage in front of the brainstem. **(B)** CTA showed a fusiform aneurysm (arrow) on the left VA. **(C)** DSA of the left VA showed the aneurysm (arrow) above PICA origin. **(D)** Three-dimensional DSA showed the aneurysm (arrow), and the contralateral VA was normally developed. **(E)** Unsubtracted DSA showed that the aneurysm was treated with PAO, and the PAO was intact. **(F)** Half-year follow-up DSA showed no recurrence of the aneurysm.BA, basilar artery; CT, computed tomography; CTA, computed tomography angiography; DSA, digital subtraction angiography; L, left; PICA, posterior inferior cerebellar artery; VA, vertebral artery.

**Figure 6:**
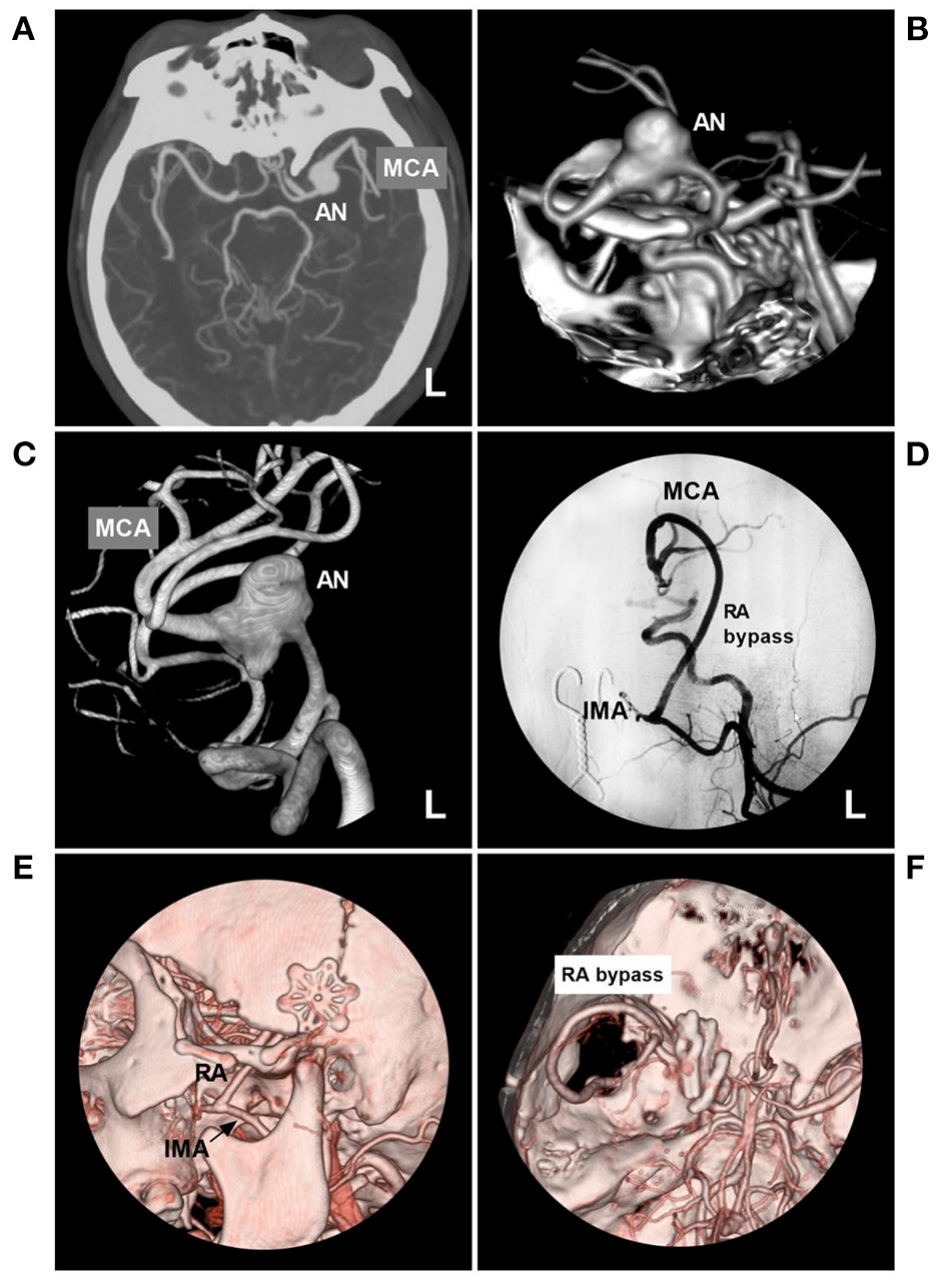
PAO with the assistance of high-flow bypass in a circumferential AN of the MCA trunk. **(A,B)** Maximum-intensity projection **(A)** and three-dimensional reconstruction **(B)** of CTA showed a left circumferential AN of the MCA trunk (AN). **(C)** Three-dimensional DSA of the left ICA clearly showed the AN. **(D)** Left DSA showed that high-flow bypass was performed between the IMA and MCA beyond the AN with the RA graft. **(E,F)** Follow-up CTA showed the bypass. **(C)** the RA was anastomosed to the IMA trunk (arrow). **(F)** the RA was anastomosed to the MCA, and the AN was trapped by multiple clips. AN, aneurysm; CTA, computed tomography angiography; DSA, digital subtraction angiography; MCA, middle cerebral artery; ICA, internal carotid artery; IMA, internal maxillary artery; L, left; RA, radial artery; PAO, parent artery occlusion.

**Figure 7:**
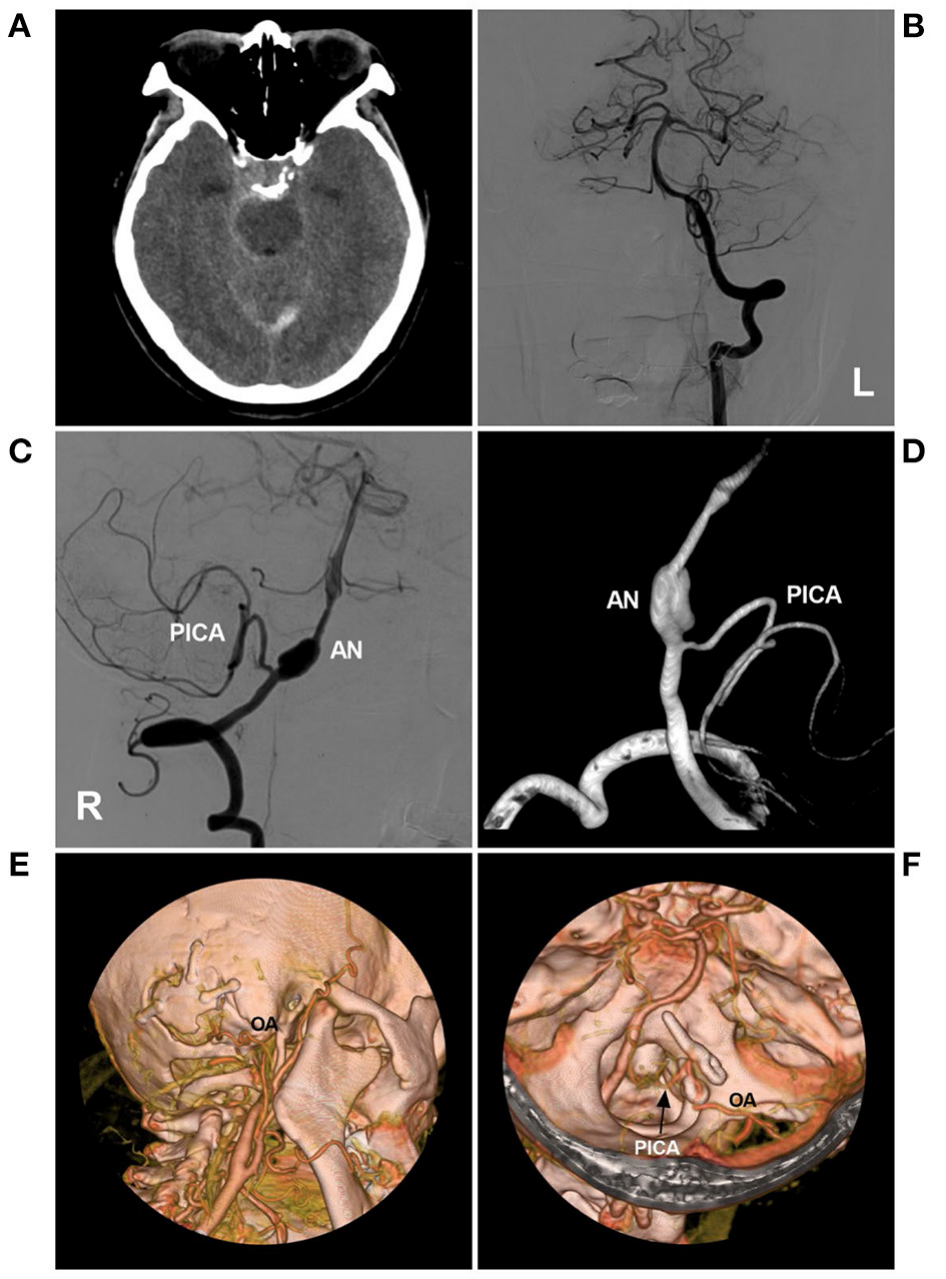
PAO with the assistance of low-flow bypass in a VA fusiform AN. **(A)** CT showed subarachnoid hemorrhage around the brainstem. **(B)** DSA of the left VA showed that the VA was normal. **(C,D)** DSA (C) and three-dimensional DSA **(D)** of the right VA showed the fusiform AN above the origin of the PICA. **(E,F)** CTA showed PAO under the assistance of low-flow bypass. **(E)** the OA entered the cranium through the bone window. **(F)** the OA was anastomosed with the PICA trunk (arrow), and the AN was trapped with the clips. AN, aneurysm; CT, computed tomography; CTA, computed tomography angiography; DSA, digital subtraction angiography; OA, occipital artery; PICA, posterior inferior cerebellar artery; L, left; PAO, parent artery occlusion; R, right; VA, vertebral artery.

Typical cases treated with PAO are shown in Figures 4–7. In the case presented in Figure 4, the giant IFCA with thrombosis grew progressively, and the PAO indication was clear. In the case presented in Figure 5, the acute dissection ruptured, and urgent EVT PAO was indicated. In the case presented in Figure 6, the IFCA has a daughter sac, which is dangerous; thus, clipping PAO with a high-flow bypass was performed. In the case presented in Figure 7, the acute dissection was too close to the posterior inferior cerebellar artery (PICA). The coiling PAO was too dangerous for the PICA; thus, clipping PAO with the occipital artery to the PICA was a good choice. The cases in Figures 4–7 had a good prognosis.

## Open Surgical Reconstruction

IFCAs can be treated with open surgical reconstruction and are never easy to perform because the entire arterial wall is circumferentially incorporated into the aneurysm, partial calcifications, and/or thrombosis as well as previous coiling (14, 69, 70). The outcome of the surgical procedure depends on adequate collateral circulation and the preoperative clinical grade. In addition, the expertise of the surgeon also influences the success rate (14).

In cases of adequate collateral supply, IFCAs, especially those with an outward weak point, can be treated by partial clipping or followed by wrapping with encircled aneurysm clips or T-bar clips (71, 72). In some cases, clipping assisted with wrapping using temporalis fascia or Gore-Tex material is more reasonable (73, 74). In cases of poor collateral supply, bypass assistance is needed when surgical reconstruction may reduce the blood flow of the parent artery (75).

**Figure 8:**
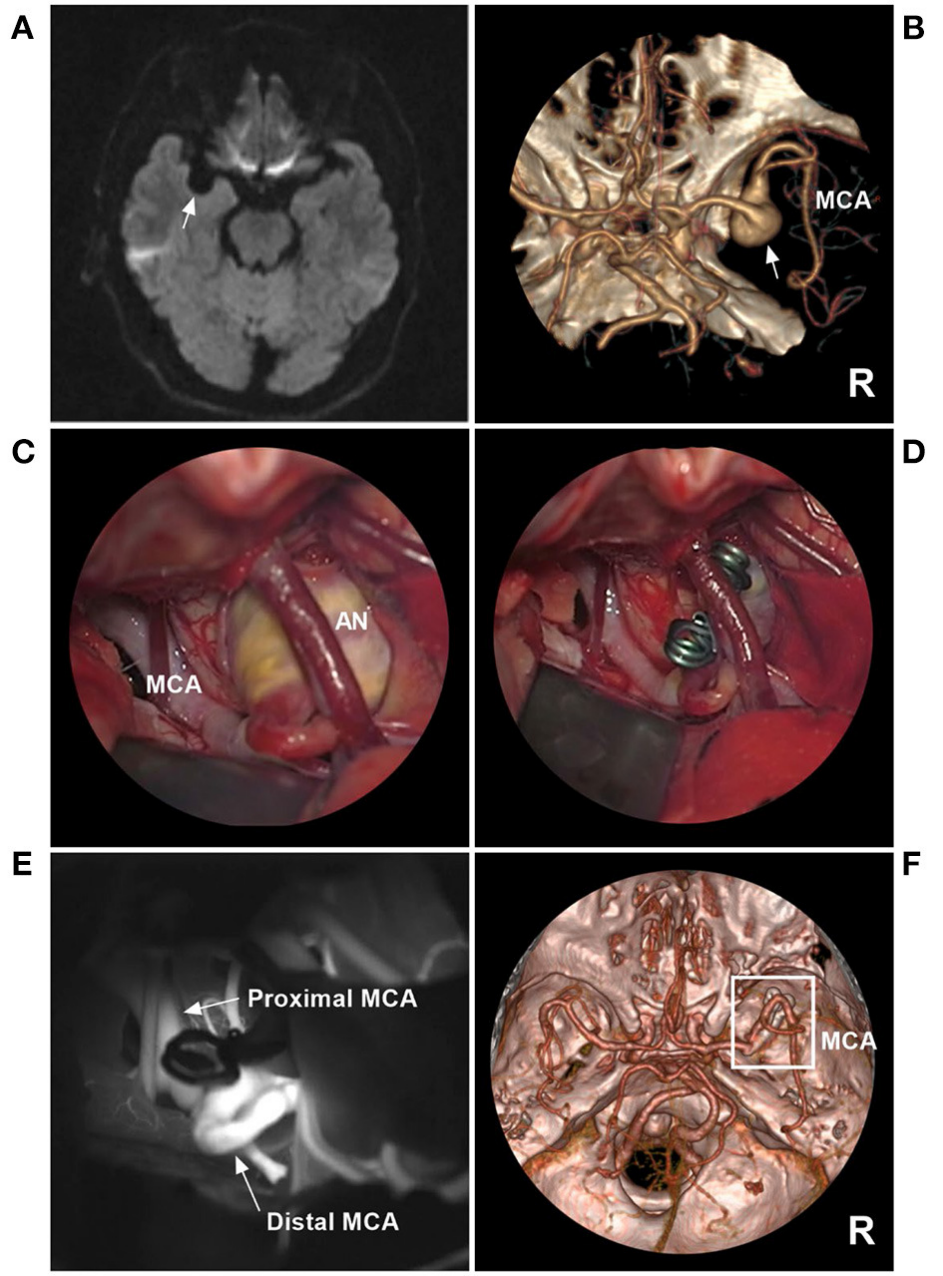
Direct clipping of a fusiform AN of the MCA trunk. **(A)** MRI showed a fusiform low-signal lesion (arrow) in the Sylvian fissure. **(B)** CTA showed that the right AN was a fusiform without a neck (arrow). **(C)** The operative image before clipping revealed the AN, and a vein crossed the AN. **(D)** The operative image showed that the AN was clipped. **(E)** Fluorescence imaging showed that the proximal and distal MCAs were unrestricted. **(F)** Follow-up CTA showed that the AN (frame) was clipped completely. AN, aneurysm; CTA, computed tomography angiography; DSA, digital subtraction angiography; MCA, middle cerebral artery; MRI, magnetic resonance imaging; R, right.

In addition, the hemodynamics in some IFCAs are intriguing. When the surgery slows and/or reverses blood flow, even if IFCAs are intact, they can occlude due to later spontaneous thrombosis (76). Typical cases treated with direct clipping are shown in Figure 8. In this case, the MCA IFCA was chronic and unruptured. To prevent progressive growth, direct clipping was performed without complications. Good prognosis is associated with the location of the IFCA. The IFCA was located in the distal MCA trunk, and the number of perforating arteries was small. In addition, the operating space was sufficiently exposed, so clipping was safe.

## Targeted Embolization

For large IFCAs, a conventional self-expandable stent assisted with coiling cannot completely obliterate IFCAs, especially those with branches arising from the aneurysmal wall (77). However, the technique is still useful for target embolization, which targets weak points or ruptured blebs by coiling assisted by conventional stents, especially for acute IFCAs located in the MCA and BA trunk (78, 79).

**Figure 9:**
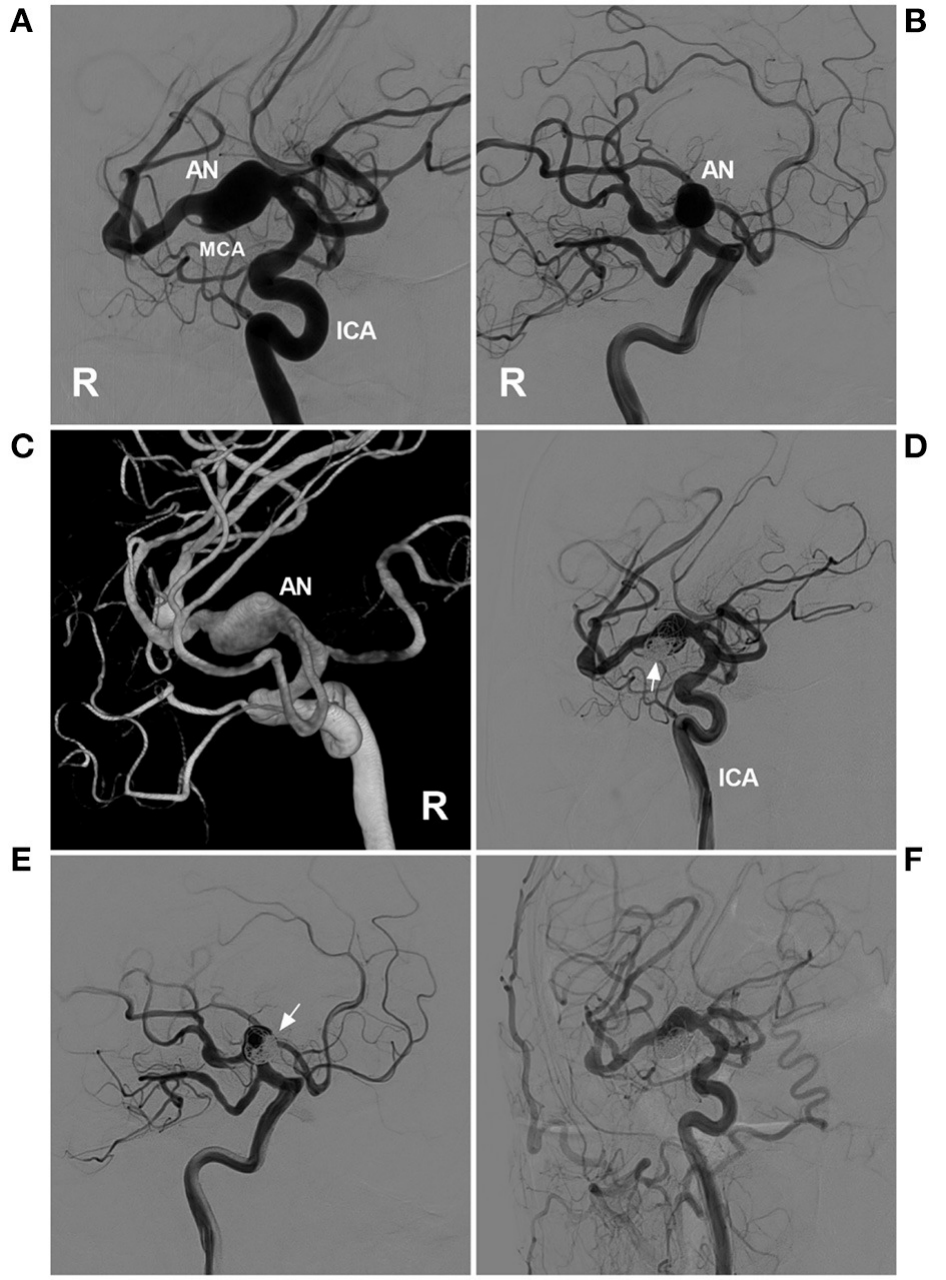
Targeted coiling for a fusiform AN of the MCA trunk. **(A,B)** DSA of the right ICA showed a fusiform AN. **(A,B)** show different projection degrees. **(C)** Three-dimensional DSA of the ICA revealed the fusiform AN more clearly. **(D,E)** DSA of the ICA showed that the AN was coiled by targeting the lower part of the aneurysm under stent assistance (arrows). **(D,E)** show the different projecting degrees. **(F)** Follow-up DSA of the common carotid artery indicating satisfactory coiling. AN, aneurysm; DSA, digital subtraction angiography; ICA, internal carotid artery; MCA, middle cerebral artery; R, right.

**Figure 10:**
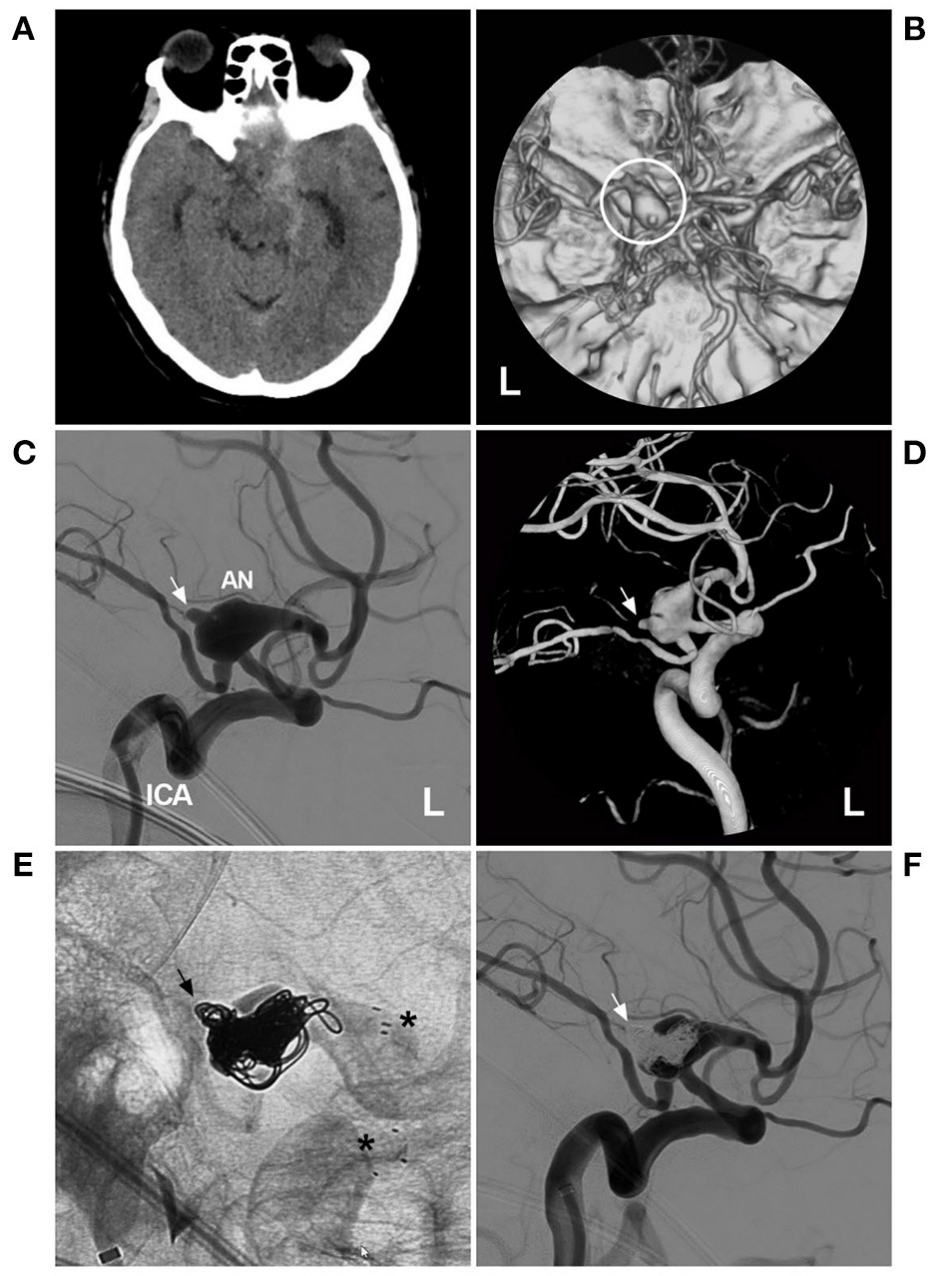
Targeted coiling for a circumferential AN of the PcomA. **(A)** CT showed right subarachnoid hemorrhage around the brainstem and in the Sylvian fissure. **(B)** CTA showed a large AN of the left PcomA (circle). **(C,D)** DSA **(C)** and three-dimensional DSA **(D)** revealed the circumferential AN of the left PcomA segment, with a ruptured bleb on the top (arrow). **(E)** X-ray film showed that the AN had been embolized with stent assistance (asterisks) and that the ruptured bleb had been filled by coils (arrow). **(F)** DSA indicated that the ruptured bleb had been satisfactorily treated by targeted embolization (arrow). AN, aneurysm; CT, computed tomography; CTA, computed tomography angiography; DSA, digital subtraction angiography; L, left; PcomA, posterior communicating artery.

Targeted embolization can be useful as temporary wall reinforcement while awaiting sequent treatment, such as FD deployment or open surgery with bypass (33). The residual part following EVT may be stable for short periods of time or represent the last resort in elderly individuals (80). Targeted embolization can be expanded in IFCAs with incorporated branches. To preserve the branch, targeted embolization may be performed (81). Typical cases treated with targeted embolization are shown in Figures 9, 10. In the case presented in Figure 9, the IFCA ruptured and formed a bleb on top of the IFCA. Thus, the target coiling aimed at the bleb was reasonable. In the case presented in Figure 10, the MCA IFCA was chronic and unruptured. To prevent progressive growth, the treatment was reasonable because the perforating arteries of the MCA mainly originated from the upper region. Thus, target coiling aimed at the upper region was safe.

## Complications and Prognosis

### Complications

Open surgery and EVT have proven to be successful for IFCAs (30). However, for IFCAs that are difficult to deal with or overcome, treatment can cause catastrophic consequences, including severe disability and even death. Complications can be mainly classified as ischemic and hemorrhagic complications (39, 82).

Ischemic complications mainly arise from the occlusion of branches around the IFCAs and insufficient collateral circulation (83). For instance, in a study of EVT in VA IFCAs, perforating artery ischemia was diagnosed in 9.6% of cases, and spinal cord infarction was diagnosed in 2.7% of cases (84). Due to the higher metal coverage, FD deployment has a higher rate of ischemic complications (85). To reduce ischemic complications, LEO stents (Balt Extrusion, Montmorency, France) with 14% metal coverage or LVIS Blue (MicroVention, Tustin, California, USA) with 22–28% metal coverage can be used with the help of a flow-diverting effect in some selective IFCAs (86–89).

Hemorrhagic complications arise from the bleeding of IFCAs during or after treatment (83). The rate of rebleeding was 4.5% in a study of IFCA clipping (71). In reconstructive EVT for ruptured IFCAs, the overall rebleeding rate was 9% (22). When treating IFCAs, PAO can result in intraoperative aneurysm rupture (59). FD deployment can result in early and late bleeding (90–93).

### Prognosis

Both surgery and EVT have proven to be successful (94). In the report of Barletta et al., 77% of surgical procedures presented good outcomes, 91.5% of reconstructive EVTs presented a good outcome, and 79.6% of deconstructive EVTs presented a good outcome (95). IFCAs in different locations may affect the outcomes. In the study by Anson et al., anterior circulation aneurysms had better outcomes than posterior circulation aneurysms with good outcomes noted in 90 and 65% of the cases, respectively (96). In addition, the combination of both surgery and EVT for IFCAs yielded an overall good clinical outcome in 77.1% of cases (64).

## Therapeutic Prospects of IFCAs

Open surgery presented worse outcomes than EVT for IFCAs (95). In a Canadian study, Drake and his group reported that the surgical approach for IFCAs was unsatisfactory and failed to solve the problem over a 1-year period (31, 97). Therefore, EVT represents the best advance in the treatment of IFCAs, particularly since the invention of FDs. Clearly, FD has a good future, and all open surgeries, including complex (non-durable) bypass, trapping, and clipping, will no longer be used. The challenge in the endovascular field in the next decade will be to design new longer, larger, and easier-to-deploy endovascular implants as well as to improve antithrombotic management, which will offer good results for IFCA treatment.

In addition, based on the pathology of genetic mutations of IFCAs, gene treatment may be expected in the future (98).

## Summary

IFCAs located on the main trunk are rare and difficult to manage. Large or symptomatic IFCAs are associated with increased mortality and rebleeding rates. When treatment is necessary, multiple strategies can be chosen, including deconstructive and reconstructive methods via both open surgery and/or EVT. Currently, FD has revolutionized the treatment of IFCAs. Despite complications, both surgical treatment and EVT are effective options for appropriately selected cases. Given the development of endovascular implants, EVT will have better prospects than open surgery in the future.

## Limitations

Currently, the definition of IFCA is confusing, and many entities with different pathologies are classified as IFCAs based on fusiform morphology. Therefore, the clinical characteristics and therapeutic prognosis of IFCAs are heterogeneous. In addition, most of the data in our review were obtained from a PubMed search, so all of the literature is not included in this review. Moreover, given the limitations of the author's understanding of IFCAs, this review may not be complete.

## Author Contributions

JY contributed to the conception, design of the manuscript, and critically revised the manuscript. YG and YS wrote the manuscript. KH and YS collected the medical records of the patients. All authors approved the final version of this manuscript.

## Conflict of Interest

The authors declare that the research was conducted in the absence of any commercial or financial relationships that could be construed as a potential conflict of interest.
